# Wnt-5a-regulated miR-101b controls COX2 expression in hippocampal neurons

**DOI:** 10.1186/s40659-016-0071-x

**Published:** 2016-02-19

**Authors:** Juan Francisco Codocedo, Nibaldo C. Inestrosa

**Affiliations:** Departamento de Biología Celular y Molecular, Facultad de Ciencias Biológicas, Centro de Envejecimiento y Regeneración (CARE), Pontificia Universidad Católica de Chile, Santiago, Chile; Faculty of Medicine, Center for Healthy Brain Ageing, School of Psychiatry, University of New South Wales, Sydney, Australia; Centro de Excelencia en Biomedicina de Magallanes (CEBIMA), Universidad de Magallanes, Punta Arenas, Chile; CARE, Biomedical Research Center, Pontificia Universidad Católica de Chile, Av. Alameda 340, 8331150 Santiago, Chile

## Abstract

**Background:**

Wnt-5a is a member of the WNT family of secreted lipoglycoproteins, whose expression increases during development; moreover, Wnt-5a plays a key role in synaptic structure and function in the adult nervous system. However, the mechanism underlying these effects is still elusive. MicroRNAs (miRNAs) are a family of small non-coding RNAs that control the gene expression of their targets through hybridization with complementary sequences in the 3′ UTR, thereby inhibiting the translation of the target proteins. Several evidences indicate that the miRNAs are actively involved in the regulation of neuronal function.

**Results:**

In the present study, we examined whether Wnt-5a modulates the levels of miRNAs in hippocampal neurons. Using PCR arrays, we identified a set of miRNAs that respond to Wnt-5a treatment. One of the most affected miRNAs was miR-101b, which targets cyclooxygenase-2 (COX2), an inducible enzyme that converts arachidonic acid to prostanoids, and has been involved in the injury/inflammatory response, and more recently in neuronal plasticity. Consistent with the Wnt-5a regulation of miR-101b, this Wnt ligand regulates COX2 expression in a time-dependent manner in cultured hippocampal neurons.

**Conclusion:**

The biological processes induced by Wnt-5a in hippocampal neurons, involve the regulation of several miRNAs including miR-101b, which has the capacity to regulate several targets, including COX-2 in the central nervous system.

## Background

The Wnt proteins constitute a large family of cysteine-rich secreted glycoproteins, which are present in all animal species. The genome of mice and humans, has 19 independent genes which are expressed in a tissue-specific form and also dependent on the development [1]. Wnts have been implicated in several cellular processes, such as cell proliferation, migration, polarity and cell fate specification [2, 3]. Moreover, the deregulation of Wnt signaling is related with several diseases, including autism [4, 5], schizophrenia [6, 7] and Alzheimer’s disease [7, 8]. Wnt ligands couple to various receptors and thereby activate different signaling pathways. On the basis of early studies, these pathways have been classified as either canonical (β-catenin-dependent) or non-canonical (β-catenin-independent) signaling pathways. However, this classification can only serve as a rough guide, as various divergent pathways has been described in different cellular contexts [9].

The role for Wnt signaling in synaptic formation and function has been clearly established [10–12]. In fact, we described that Wnt-5a, which preferentially activates non-canonical pathways [9], exerts important effects in the postsynaptic region of central synapses. Wnt-5a stimulation increases the postsynaptic density protein 95 (PSD-95) clustering [13] and increases the density of dendritic spines [14]. In hippocampal slices, Wnt-5a enhances long-term potentiation (LTP) modulating synaptic activity and plasticity [15, 16]. These findings strongly suggest that Wnt-5a regulates the assembly and function of the excitatory postsynaptic region of central synapses [17]. However, the mechanism underlying these effects is still elusive.

MicroRNAs (miRNAs) are a class of small non-coding RNAs that regulate the local translation of dendritic mRNAs, affecting the morphology and function of dendritic spines [18]. MiRNAs control gene expression through specific base pairing between the 3′ UTR of mRNA and the miRNA “*seed”* region at the 5′ end [19]. We recently describe the miRNA biogenetic pathway in recent reviews [20, 21]. Briefly, canonical miRNAs are transcribed as primary miRNAs (pri-miRNAs, a long stem-loop precursor of several hundred nucleotides) which is cropped by the Microprocessor complex, composed by DiGeorge Syndrome Critical Region 8 (DGCR8) and Drosha, a double-stranded RNA binding protein and an RNase III enzyme, respectively [22]. The resulting pre-miRNA (~70 nt in length) is exported to the cytoplasm by Exportin-5 in a GTP-dependent fashion [23]. In the cytoplasm, pre-miRNA is cleaved into a ~22 nt mature miRNA duplex by Dicer, a second RNAse III enzyme. One strand of the mature miRNA duplex is loaded into the miRNA-induced silencing complex (miRISC) with members of the Argonaute family of proteins, producing a functional complex for targeting mRNA via direct base pairing [24]. The resulting miRNA/mRNA hybrids alter protein expression of the targeted mRNA by different mechanisms, including translational repression or mRNA degradation [25]. Some miRNAs have alternative biogenesis process, because they can bypass the action of some processing complexes. This is mainly due to structural differences in the precursors, which allow processing by other protein complexes such as the spliceosome. These exceptions are known as non-canonical pathways [26].

Interestingly, several reports have shown that miRNAs are downstream mediators of different extracellular stimuli, such as glutamate [27], dopamine [28], serotonin [29] and brain-derived neurotrophic factor (BDNF) [30], contributing to the induction and consolidation of plastic changes triggered by these synaptogenic factors.

In the present study, we provided evidence for a new mechanism underlying the neuronal effects of Wnt-5a, describing a number of miRNAs responsive to this ligand in hippocampal neurons. We focused on miR-101b, the most affected miRNA through Wnt-5a signaling, and their target COX2, an inducible enzyme that converts arachidonic acid to prostanoids, and has been related to the injury/inflammatory response [31, 32], and more recently to neuronal plasticity [33–36]. The downregulation of miR-101b could contribute to the increase in COX2 expression observed during prolonged exposure to Wnt-5a, revealing a new effector of Wnt signaling in hippocampal neurons.

## Methods

### Ethics statement

Sprague-Dawley rats were housed in the University Animal Facility and handled according to the guidelines outlined and approved through the Institutional Animal Care and Use Committee at the Faculty of Biological Sciences of the Pontificia Universidad Católica de Chile, and following the guidelines of the American Physiological Society Rockville, MD, USA.

### Primary culture of rat hippocampal neurons

Rat hippocampal cultures were prepared as previously described [37, 38]. Primary hippocampal neurons were obtained from 18-day-old Sprague-Dawley rat embryos and maintained in Dulbecco’s modified Eagle’s medium (DMEM) supplemented with 10 % horse serum for 2 h. The culture medium replaced with Neurobasal medium supplemented with B27, 100 µg/ml streptomycin, and 100 units/ml penicillin. At 3 days in vitro (DIV), the cells were treated with 2 µM Cytosine Arabinoside C (araC) for 24 h to reduce the number of glial cells present in the culture. For the miRNA expression studies, 800,000 cells per well were seeded. For western blot (WB) analyses, 400,000 cells per well were seeded, and for immunofluorescence studies, 35,000 cells were plated per well. At 14 DIV, the neurons were stimulated with 50 µM of Foxy-5 (a mimetic formylated hexapeptide of Wnt-5a) (Genemed Synthesis, San Francisco, CA, USA) or 300 ng/mL of recombinant Wnt-5a (rWnt-5a) (R and D System, Minneapolis, MN, USA) resuspended in Neurobasal medium. Control neurons incubated with a scramble peptide in Neurobasal medium (Genemed Synthesis, San Francisco, CA, USA) for experiments with either FOXY-5 or the carrier (BSA 0.1 %) resuspended in neurobasal medium for experiments with rWnt-5a. Incubations were conducted at 37 °C.

### HT22 cell line

HT22 murine hippocampal neuronal cells were maintained in DMEM supplemented with 10 % fetal bovine serum, 100 µg/ml streptomycin, and 100 units/ml penicillin, high glucose and incubated at 37 °C under 5 % CO_2_ as previously described [39]. Transfections were performed after 2 days at approximately 60 % confluency.

### RNA extraction and Real-time PCR-based miRNA expression profiling

Total RNA extraction and subsequent enrichment of small RNAs (<200 nt) was performed using the miRVana kit (Ambion) according to the manufacturer’s instructions [40]. The quantity and purity of the RNA samples were assessed using a NanoDrop 2000 Spectrophotometer (Thermo Scientific). The integrity of the small RNAs was assessed on denaturing 15 % polyacrylamide gels.

First-strand cDNA was synthesized from 150 ng of small RNA using the RT^2^ miRNA First Strand kit (SABiosciences). RT^2^ miRNA PCR array was performed with an Mx3000p qPCR system (Stratagene). The reactions, containing 2X RT^2^ SYBR PCR master mix and diluted cDNA in a final volume of 25 μl, were amplified at 95 °C, 15 s; 60 °C, 30 s; 72 °C, 30 s for 40 cycles. The miRNA input was normalized to endogenous controls (Rnu6, U87, 4.5S-V1 and Y1), and the data analysis was performed using the web-based software package for the miRNA PCR array system using the comparative ΔΔCt method [41]. The fold-change was calculated for each miRNA from cells treated with Foxy-5 compared with control, represented as 2^(−ΔΔCt)^, and the results were expressed as the fold-regulation by taking the negative inverse of any number less than 1, changing the fractional number into a whole number.

To evaluate the expression of COX-2 mRNA, 20 μg/μl of total RNA was used for cDNA synthesis using the GoScript Reverse Transcription System (Promega). The determination of the mRNA abundance of specific genes was assessed through quantitative PCR (qPCR), using the StepOne 48-well Real-Time PCR System (Applied Biosystems, Carlsbad, CA, USA) and Fast SYBR Green Master Mix (Applied Biosystems) reagent for amplification, using 4 μl cDNA, 4 μl DEPC water, 10 μl Master Mix, 1 μl Reverse Primer, and 1 μl Forward Primer in a total volume of 20 μl/well. The housekeeping gene was GAPDH (forward primer: 5′-CACGGCAAGTTCAACGGC-3′; reverse primer 5′-GGTGGTGAAGACGCCAGTA-3′) at an annealing temperature of 60 °C. The following primer sequences were used for COX-2: forward primer: 5′-TGTATGCTACCATCTGGCTTCGG-3′, reverse primer 5-′GTTTGGAACAGTCGCTCGTCATC-3′.

### Bioinformatics

For computational prediction of the miRNA targets, we used the TargetScan web platform [42], which predicts biological targets of miRNAs by searching for the presence of conserved 8- and 7-mer sites that match the seed region of each miRNA. In addition, TargetScan examines the binding sites for thermodynamic stability using RNAfold from the Vienna RNA Package [43]. For testing enrichment in specific KEGG pathways of miRNA targets, we used DIANA-mirPath [44]. The software performs an enrichment analysis of multiple miRNA target genes comparing each set of miRNA targets with all known KEGG pathways. The combinatorial effect of co-expressed miRNAs in the modulation of a given pathway was considered through the simultaneous analysis of multiple miRNAs.

### miR-101b gain-of-function

For miR-101b gain-of-function, we used mirVana miRNA mimic (Life Technologies), which represents a partially double-stranded RNA that mimics endogenous precursor miRNA and is processed to form an active miRNA molecule that targets specific mRNAs [45]. For miR-101b gain-of-function in HT-22 cells, Lipofectamine 2000 reagent (Invitrogen, Karlsruhe, Germany) was used according to the manufacturer’s protocol. At 48 h post-transfection, cells were used for western blotting (WB) or immunofluorescence analysis. For controls conditions HT22 cells was transfected with mirVana miRNA Mimic Negative Control #1 (Life Technologies, Carlsbad) which correspond to a random sequence miRNA mimic molecule that not produce identifiable effects on known miRNA function.

### Western blot analysis

The extraction of total protein from cell culture of hippocampal neurons and immunoblot analysis were performed as previously described [46, 47]. The following primary antibodies were used: rabbit anti-COX-2 (1:1000; ABCAM) and anti GAPDH (1:10,000, Santa Cruz). Primary antibodies were recognized using either a horseradish peroxidase (HRP)-conjugated goat anti-rabbit antibody (1:7000, Thermo Scientific) or an HRP-conjugated rabbit anti-mouse antibody (1:7000, Thermo Scientific). The secondary antibodies were detected through enhanced chemiluminescence using the ECL Plus WB detection system (GE Healthcare). Densitometric analysis was performed using NIH ImageJ software.

### Immunofluorescence analysis

Immunofluorescence studies were performed as previously described [46, 47]. Briefly, after stimulation, the cells were rinsed twice in ice- cold PBS and fixed with a freshly prepared solution of 4 % paraformaldehyde and 4 % sucrose in PBS for 20 min at 4 °C and permeabilized with 0.2 % Triton X-100 for 5 min in PBS at room temperature. After several rinses in ice-cold PBS, the cells were incubated in 1 % BSA in PBS (blocking solution) for 30 min at room temperature, followed by an overnight incubation at 4 °C with primary antibodies. The cells were extensively washed with PBS and subsequently incubated with Alexa-conjugated secondary antibodies for 60 min at 37 °C. The coverslips were mounted in mounting medium, and image stacks were collected in 0.25 µm z-step sizes using an Olympus LSM Fluoview 1000 confocal microscope. The images in the figures are maximum intensity projections to obtain high contrast images, but the quantifications were made over average intensity projections. An outline was drawn around each cell and total fluorescence measurements were performed with NIH ImageJ software.

### Statistical analysis

All data were analyzed statistically with Prism 5 (Prism GraphPad Software, GraphPad Software Inc., La Jolla, CA, USA) using the Mann-Whitney *U* test or one-way ANOVA, followed by Bonferroni-corrected pairwise comparisons. The error bars indicate SEM. A p < 0.05 was considered statistically significant.

## Results

### Wnt-5a signaling regulates the expression levels of miRNAs in hippocampal neurons

**Fig. 1:**
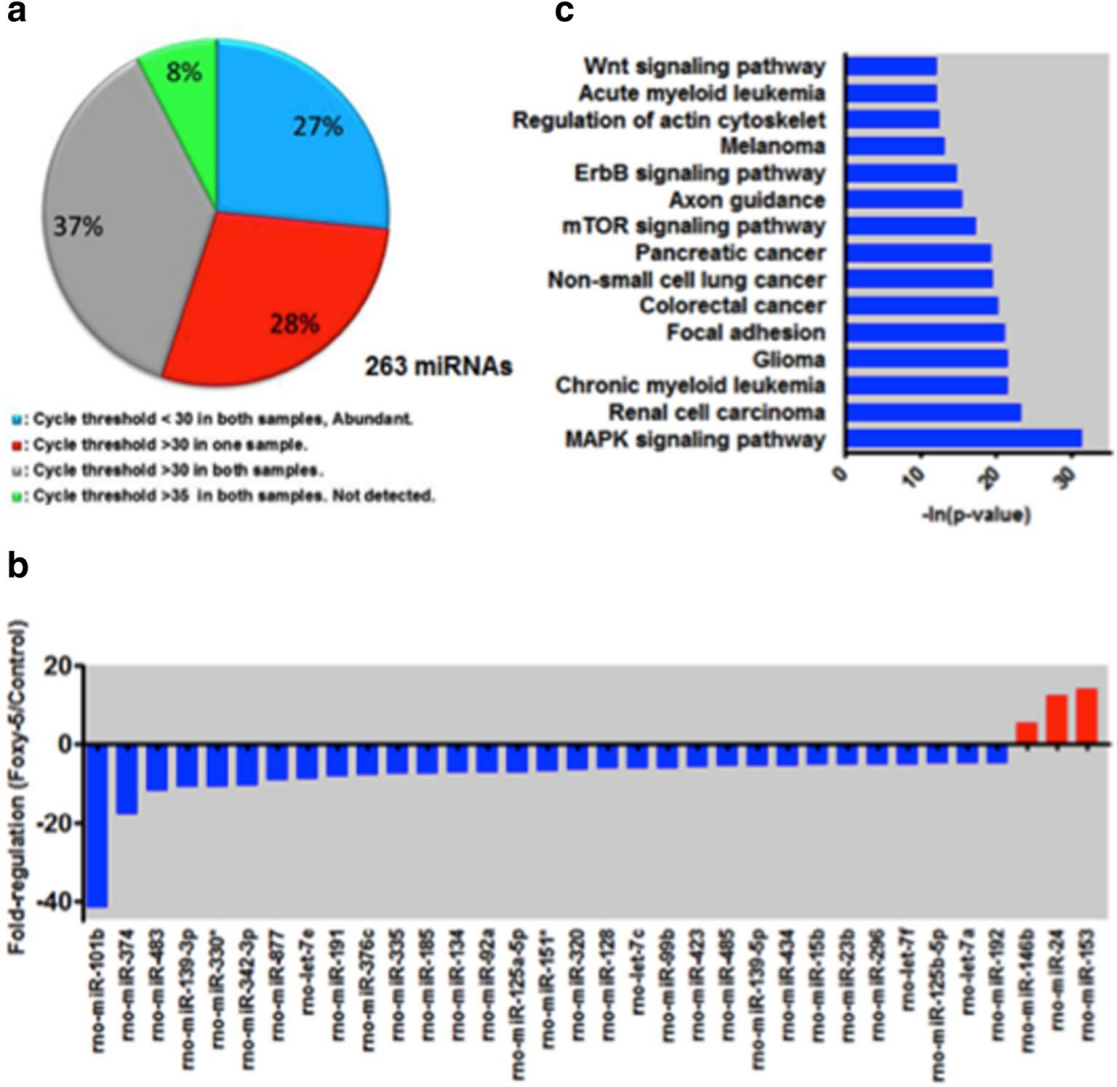
Foxy-5, a mimetic peptide of Wnt-5a, regulates miRNA expression levels in hippocampal neurons. **a** The *pie chart* shows the percentage expression of 263 rat miRNAs in hippocampal neurons treated with Foxy-5 and control (n = 3, each). **b** Fold-regulation of miRNAs expression in neurons treated with Foxy-5 (p < 0.05, n = 3). Among the 34 significantly modulated miRNAs, miR-101b was the most affected. **c** The top KEGG pathways of biological function of the targets of downregulated miRNAs after Foxy-5 treatment

Various functions of different cell types in the mammalian brain suggest that some miRNAs are differentially expressed in glia and neurons in response to the same stimuli [48, 49]. To determine whether Wnt-5a modulates neuronal miRNAs, we profiled miRNAs in cultured hippocampal neurons with a low glial content (see “Methods” section). These cultures were treated with Foxy-5, a mimetic formylated hexapeptide of Wnt-5a, extensively used for the reproduction of the biological effects of this ligand [50–53]. Foxy-5 mimics the full Wnt-5a molecule in cultures of hippocampal neurons [13, 16, 54, 55]. Among the 264 miRNAs tested after 1 h of stimulation, 8 % miRNAs were not detected, 37 % miRNAs showed low detection and 27 % miRNAs were abundantly expressed. The remaining 28 % miRNAs were abundantly detected in at least one of the experimental conditions (control or treated) (Fig. 1a). Consistent with the suggestions of the supplier, for the next analysis, we used only a fraction of miRNAs abundantly detected in at least one of the samples (cyan and red groups in Fig. 1a). In addition, to be considered as a regulated miRNAs, a minimum of fivefold regulation was used as an inclusion criteria as previously described [48]. In this fraction, we observed significant changes in 34 miRNAs (p < 0.05). Three of these miRNAs showed increased expression (miR-24, -146b and -153) and 31 miRNAs showed decreased expression (Fig. 1b).

### Biological function of the miRNAs modified by Wnt-5a signaling

Using the online software TargetScan, we predicted a list of target genes for the miRNAs genes modulated through Foxy-5. To understand the functional significance of these targets, we used the online software, Diana miRpath, which identifies biological processes downstream of miRNAs altered through Wnt-5a signaling. The major biological functional categories targeted by downregulated miRNAs are enriched in pathways associated with cancer. Other pathways were associated with synaptic processes, such as MAP-kinase signaling, focal adhesion, mTOR signaling, axon guidance, regulation of actin cytoskeleton and Wnt signaling (Fig. 1c). Altogether, these results suggest the possible roles and mechanisms of these differentially expressed miRNAs and their targets in the presence of the Wnt-5a ligand.

### Mir-101b gain-of-function downregulates COX2 expression

**Fig. 2:**
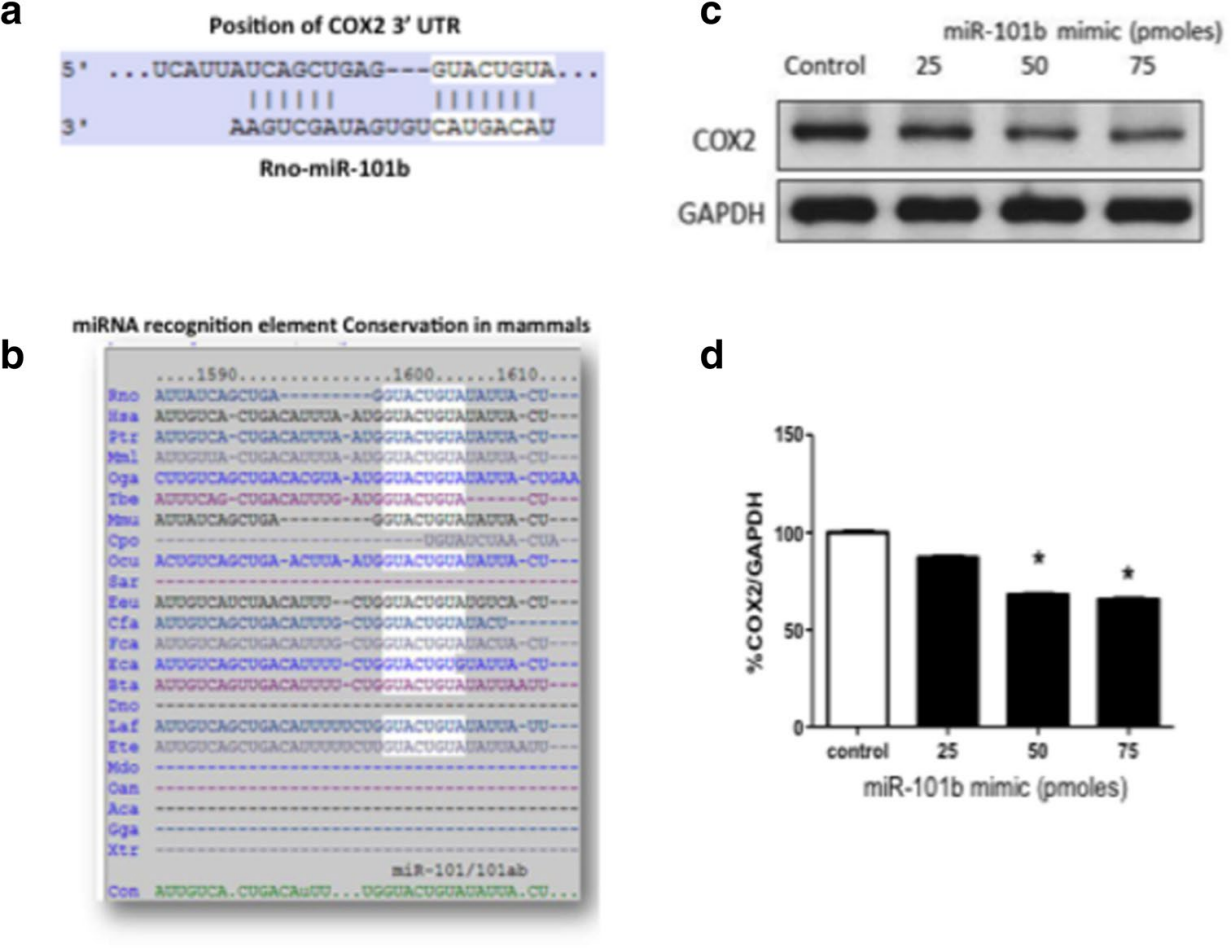
COX2 is a target of downregulated miR-101b in hippocampal neurons. **a** Complementarity of the sequences between miR-101b and the COX2 3′-UTR of rat genome obtained from TargetScan. **b** The predicted binding site for the seed sequence of miR-101b in the 3′UTR of COX2 is conserved among several species. **c** Endogenous levels of COX2 are significantly decreased through gain-of function of miR-101b mimics in HT22 cells **d** Densitometric analysis of the WB shown in c (*p < 0.05, n = 4)

Considering that Wnt-5a signaling generates a significant decrease in the levels of miR-101b (Fig. 1b), we reasoned that the levels of some of the predicted targets could be increased in the presence of Wnt-5a. Among these targets, we focused on COX2, an enzyme expressed in discrete populations of neurons and is enriched in the cortex and hippocampus [56] and has been implicated in brain functions and in neurologic disorders, including Alzheimer’s disease [57]. The miRNAs recognition element (MRE) in the 3′UTR of COX2 is a canonical binding site (8-mer) broadly conserved among mammals (Fig. 2a, b). To validate the in silico prediction of COX2 as a target of miR-101b, we introduced a miRNA mimic into HT22 cells and evaluated the expression of endogenous COX2 through WB (Fig. 2c). It is apparent that the increase of miR-101b decreases the level of COX2 in a dose-dependent manner (Fig. 2d). The same effects were observed in immunofluorescence experiments in HT22 cells using the maximal efficacy concentration of miR-101b mimics (data not show). These results validate the in silico prediction of COX2 as a target of miR-101b and suggest that COX2 expression could be modulated through Wnt-5a signaling via miR-101b.

### Wnt-5a treatment increases the expression of COX2 in cultured hippocampal neurons

**Fig. 3:**
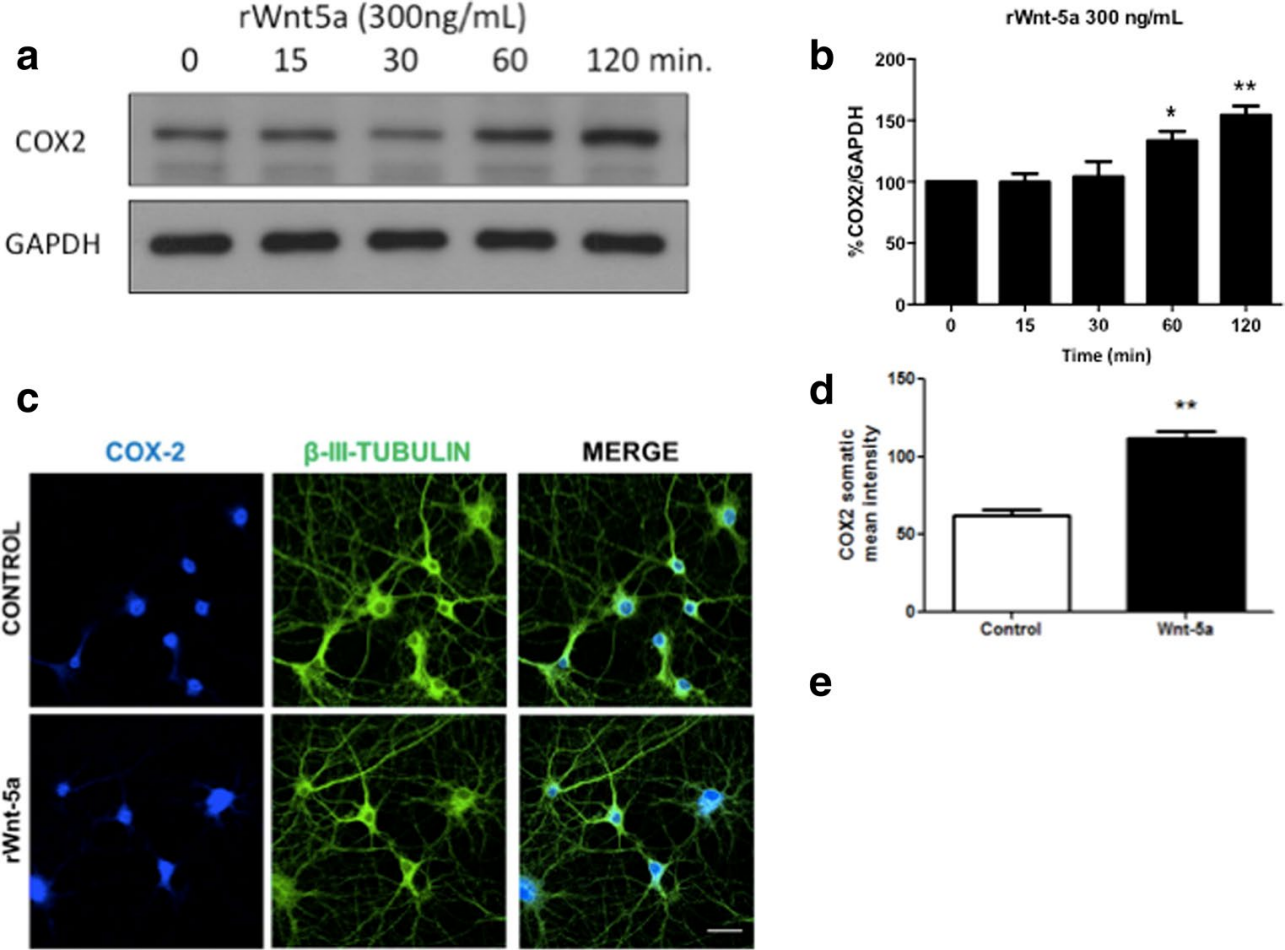
Wnt-5a treatment increases the expression of COX2 in a time-dependent manner. **a** WB analysis of COX2 levels in hippocampal neurons treated with recombinant Wnt-5a (300 ng/mL) at different time points. **b** Densitometric analysis of the WB experiments shown in **a**. The results are presented as the mean of n = 5 experiments, and the statistical analysis was performed using one-way ANOVA, followed by post hoc Bonferroni’s analysis *p < 0.05 **c** Immunofluorescence analysis of COX2 expression in hippocampal neurons. *Upper panel* Representative images of control hippocampal neurons at 14 DIV. *Lower panel* Representative images of hippocampal neurons treated with rWnt-5a (300 ng/mL) for 1 h at 14 DIV (COX2 *Blue*, β-III-Tubulin *green*. *White bar* corresponds to 20 μm). **d** Quantification of somatic mean fluorescence intensity. n = 3, and the statistical analysis was performed using the Mann-Whitney *U* test * p < 0.05. **e** Analysis of COX2 mRNA expression using quantitative real-time PCR in hippocampal neurons treated for 1 h with rWnt-5a (300 ng/mL) at 14 DIV. The results are normalized to GAPDH expression and represented as the mean of n = 3 experiments. The statistical analysis was performed using the Mann–Whitney *U* test (NS, no significant changes)

The expected outcome of a decrease in miRNA levels is that the translation of the target increases, and as a consequence, the target protein levels also might increase. Using WB analyses, we determined that treatment with Wnt-5a increases the levels of COX2 in a time-dependent manner in cultured hippocampal neurons (Fig. 3a, b). Complementary immunofluorescence studies confirm this observation, because Wnt-5a increases the somatic and dendritic signal of COX-2 at 1 h of stimulation (Fig. 3c, d). This increase is coincident with the previously described decrease in miR-101b triggered through Wnt-5a signaling activation (Fig. 1b). Using qPCR, we measured the expression of COX2 mRNA to determine whether this effect is dependent on a transcriptional mechanism. At 1 h of stimulation, we did not detect changes in the levels of COX2 mRNA expression (Fig. 3e), suggesting a post-transcriptional mechanism, consistent with the miR-101b decrease.

## Discussion

Previous studies indicated that Wnt-5a is a synaptogenic factor, whose expression increases during development; indicating that Wnt-5a plays an important role in synaptic structure and function in the adult nervous system [17, 58]. Neural factors induce plastic changes through the activation of different signaling pathways, including the modulation of specific miRNAs [59]. In the present study, we evaluated the hypothesis that treatment with Wnt-5a modulates miRNAs in hippocampal neurons and this modulation contributes to the neuronal effects previously described for the Wnt ligand through the regulation of different targets. We identified more than 30 miRNAs with differential expression after 1 h treatment with the Wnt-5a mimetic peptide. Most of the regulated miRNAs showed decreased expression, and only 3 miRNAs showed increased expression, suggesting that these two groups of miRNAs have different Wnt responsive elements in their biogenetic pathway. Further studies are necessary to determine the mechanism by which Wnt-5a controls the expression of miRNAs in hippocampal neurons.

To understand the role of the miRNAs regulated through Wnt-5a, we used bioinformatics tools to obtain the predicted targets and determine the potential roles for these genes in biological pathways using the KEEG database. The top KEGG pathways of biological function for the targets of downregulated miRNAs showed enrichment in cancer processes. This finding is important, since Wnt-5a is upregulated in several types of cancer. For example, Wnt-5a is increased in melanoma [60], colorectal cancer [61], pancreatic cancer [62], non-small cell lung cancer [63, 64], renal cell carcinoma [64] and glioma [65, 66]. The only exception to this, is myeloid leukemia, in which Wnt-5a is downregulated and functions as a tumor suppressor [67]. This correlation suggests an association between the in silico prediction and the known effects of Wnt-5a. However, the enrichment analysis showed some pathways with a specific role in the regulation of synaptic plasticity process, such as the MAPK signaling, focal adhesion, axon guidance, regulation of actin cytoskeleton and Wnt signaling, which is consistent with our hypothesis.

MiR-101b was the most affected miRNA in hippocampal neurons after Wnt-5a signaling activation. This miRNA has been described as a tumor suppressor that inhibits the expression of oncogenes, such as RAB GTPase 5A (RAB5A) [68] and enhancer of zeste 2 (EZH2) [69]. A number of studies have shown that the expression of miR-101b is significantly decreased in multiple types of cancer, such as colon cancer [70–72], in which, as previously noted, Wnt-5a levels are increased. The same relationship has been observed in Alzheimer’s disease, where the levels of miR-101b are reduced [73–75], but the levels of Wnt-5a are increased [76]. Since the role of miR-101b has been investigate in cancerogenesis, the roles for miRNAs in the brain have just begun to emerge. MiR-101b regulates the expression of a key protein in Alzheimer´s disease, the amyloid precursor protein (APP) [77–79], as well as other proteins involved in neurological diseases such ataxin1 [80], and the Autism protein the Fragile X Mental Retardation gene 1 (FMR1) [81], moreover, a key synaptic protein SynGAP1 [82] has been also detected in the hippocampus. Among the many targets predicted for miR-101b, we investigated COX2 for two reasons. First, the relationship between miR-101b and COX2 has been previously describe in several cancer cell lines and tumors, and we want to determine whether this regulation is conserve in hippocampal neurons and if it is regulated by Wnt-5a. Second, COX2 has been related to inflammation, synaptic plasticity and Alzheimer disease, which correspond to biological processes in which Wnt-5a is involved and plays a key role.

We validated the in silico prediction through the downregulation of the endogenous levels of COX2, using miR-101b mimics in HT22 cells. The expected outcome of a miRNA decrease is that the expression of the target proteins increases. Wnt-5a generates an increase in the protein levels of COX2 after 1 h of treatment in cultured hippocampal neurons. Immunofluorescence analysis and confocal microscopy confirm that treatment with rWnt-5a is able to induce the expression of COX-2. The localization of COX-2 in basal conditions was detected mainly in the nucleus, which is consistent with the location previously described in cortical neurons, both by electron microscopy and immune-gold techniques, which was determined at the level of the luminal surface of the nuclear membrane [83]. Besides, a weaker level of cytosolic signal was found (Fig. 3c upper panel). Treatment with rWnt-5a generated a significant increase in fluorescence intensity of COX2, which is distributed throughout the somato-dendritic compartment (Fig. 3c lower panel, d). Interestingly, the mRNA levels of COX2 were not affect at 1 h of stimulation (Fig. 3e), suggesting that the increase in their protein levels is dependent on a post-transcriptional mechanism consistent with the function of the miRNAs.

The functional consequence of COX2 increase is relate to the pro-inflammatory effect of Wnt-5a, described previously. Wnt-5a, which is expressed in astrocytes in the adult mouse brain, evokes a microglia pro-inflammatory transformation characterized by an increase in the expression of cytokines, chemokines and metalloproteases and changes in microglial proliferation and invasiveness. In a recent study, astrocytes and microglial cells prepared from newborn C57Bl 6 mice, was treated with 300 ng/mL of rWnt-5a (same amount used in this study) which generate a modest increase in the mRNA and protein levels of COX2 after 6 h, which is increased when the amount of rWnt-5a is raised up to 1000 ng/mL [84, 85]. Interestingly, in human aortic endothelial cells (HAECs), the treatment with rWnt-5a by 1 h, generates a robust increase in the protein and mRNA of COX2, however, Wnt-5a did not up-regulate the mRNA levels of COX-2 in other cell types, such as SH-SY5Y, HeLa, HEK293T, and RAW264.7, suggesting that Wnt-5a-induced inflammatory gene expression was specific for endothelial cells [86]. The signaling mediated by Wnt ligands are highly dependent of the cellular context [87], and this could be the reason of the observed differences in the induction of COX2 in different cellular models.

At difference of other tissues, COX-2 is also constitutively expressed in brain [56], suggesting that their basal activity is involved in general cellular functions. In brain, both COX-2 mRNA and protein are express at relatively high levels in neurons involved in plastic and cognitive functions, such as those hippocampal granule cells, pyramidal cells, and cortical neurons. In fact, COX-2 protein is express at a very high level in dentate granule cells under basal conditions [56].

Basal expression of COX-2 is regulate by the synaptic activity, and its cellular expression is upregulated by a high-frequency stimulation (HFS) associated with LTP induction [56]. Moreover, COX-2 is present in neuronal dendritic spines where excitatory synapses are located [88]. These evidences imply that COX-2 plays an important role in synaptic modifications. Based in our previous work, in which Wnt-5a induces an enhancement of the synaptic structure and function, the observed increase in the COX2 levels through miR-101b, is related to their role as synaptic modulator. Additional experiments are required to determine the mechanism by which Wnt-5a regulates the expression of miRNAs, in particular miR-101b and also the physiological conditions in which the module miR-101b/COX2 contribute to the brain function regulated by the Wnt-5a ligand.

**Fig. 4:**
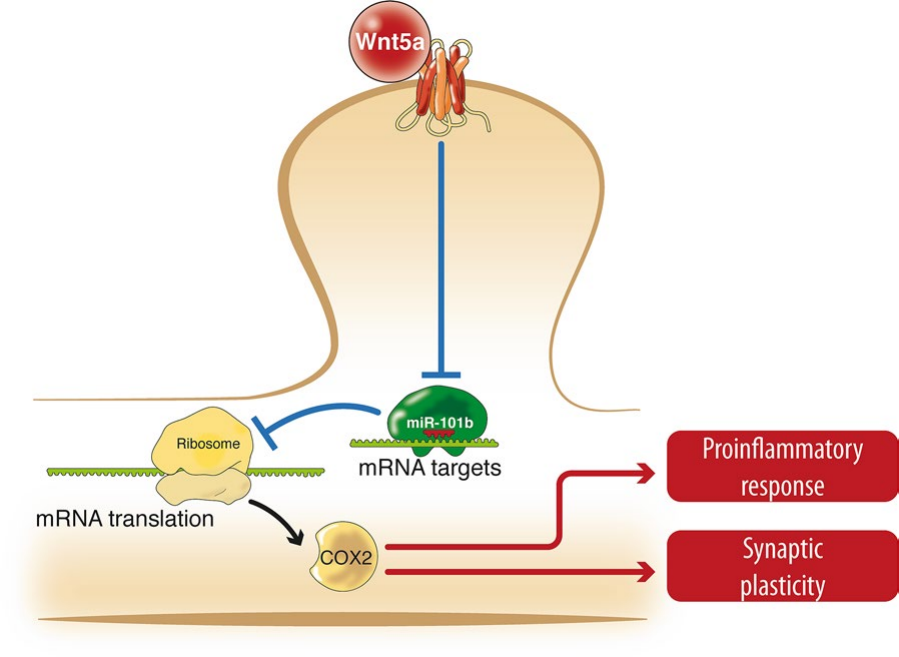
Hypothetical model for the neuronal effects of Wnt-5a through miRNAs. Wnt-5a increases the expression of COX2 through the regulation of miR-101b, which in turn could participate in biological processes well described of Wnt-5a such as a pro-inflammatory response or synaptic plasticity

Based on the results obtained in the present study, we propose a hypothetical model, in which Wnt-5a increases the expression of COX2 through the regulation of miR-101b, which in turn could participate in biological processes well described of Wnt-5a such as a pro-inflammatory response or synaptic plasticity (Fig. 4).
